# Effects of Molecular Hydrogen on Methamphetamine-Induced Neurotoxicity and Spatial Memory Impairment

**DOI:** 10.3389/fphar.2019.00823

**Published:** 2019-07-23

**Authors:** Di Wen, Rongji Hui, Jian Wang, Xi Shen, Bing Xie, Miao Gong, Feng Yu, Bin Cong, Chunling Ma

**Affiliations:** ^1^College of Forensic Medicine, Hebei Medical University, Hebei Key Laboratory of Forensic Medicine, Collaborative Innovation Center of Forensic Medical Molecular Identification, Shijiazhuang, China; ^2^College of Public Health, Hebei Medical University, Shijiazhuang, China; ^3^Department of Histoembryology, Hebei Medical University, Shijiazhuang, China

**Keywords:** molecular hydrogen, methamphetamine, spatial learning and memory impairment, mitochondrial dysfunction, endoplasmic reticulum stress, neuroinflammation

## Abstract

Methamphetamine (METH) is a highly addictive stimulant, and METH exposure can induce irreversible neuronal damage and cause neuropsychiatric and cognitive disorders. The ever-increasing levels of METH abuse worldwide have necessitated the identification of effective intervention strategies to protect the brain against METH-induced neurotoxicity. The protective effects of molecular hydrogen on oxidative stress and related neurodegenerative diseases have been recently elucidated. Herein, we investigated whether treatment with molecular hydrogen ameliorated the METH-induced neurotoxicity and spatial learning and memory impairments. Male C57BL/6 mice received four intraperitoneal METH injections (10 mg/kg, 3-h interval), and stereotypic behaviors and hyperthermia were observed. After METH treatment and behavioral observation, the mice were returned to their home cages, where they received water or hydrogen-rich water (HRW) *ad libitum* for 7 days. We found that the molecular hydrogen delivered by *ad libitum* HRW consumption significantly inhibited the METH-induced spatial learning impairment and memory loss evidenced in the Barnes maze and Morris water maze tests. Furthermore, molecular hydrogen significantly restrained the neuronal damage in the hippocampus after high-dose METH exposure. *Ad libitum* HRW consumption also had an inhibitory effect on the METH-induced increase in the expression of Bax/Bcl-2, cleaved caspase-3, glucose-related protein 78 (GRP 78), CCAAT/enhancer-binding protein homologous protein (CHOP), and p-NF-kB p65 expression and elevation of interleukin (IL)-6 and tumor necrosis factor (TNF)-α levels in the hippocampus. These are the first findings to indicate that hydrogen might ameliorate METH-induced neurotoxicity and has a potential application in reducing the risk of neurodegeneration frequently observed in METH abusers.

## Introduction

Methamphetamine (METH) is one of the most frequently abused pharmacologic psychostimulant illicit drugs with strong neurotoxic effects on the central nervous system (CNS). Long-term or high-dose use of METH causes abnormalities in the structure, chemistry, and function of brain, resulting in a number of associated medical complications and fatalities (Zhu et al., 2000; Northrop and Yamamoto, 2015; Shaerzadeh et al., 2018). Notably, METH abusers are more likely to develop schizophrenia, depression, Parkinson’s disease, and other neuropsychiatric and cognitive disorders; these are mostly attributed to METH-induced neurotoxicity (Schroder et al., 2003; Hruba et al., 2010; Kuehn, 2011).

The neurotoxic mechanism of METH is complex and involves multiple pathways (Ceccatelli, 2013; Shin et al., 2017). METH intake elicits a massive release of dopamine (DA) and excessive glutamate production in the brain, generating a large amount of reactive oxygen species (ROS), and subsequently leading to mitochondrial dysfunction and endoplasmic reticulum (ER) stress (Beauvais et al., 2011; Irie et al., 2011; Dang et al., 2018; Shin et al., 2018). METH has been shown to significantly increase the Bax/Bcl-2 ratio and to disrupt the potential of mitochondrial membranes, triggering a caspase cascade (Jayanthi et al., 2001). METH also increases the detachment of the major chaperone protein from the transmembrane ER signaling protein glucose-related protein 78 (GRP78) and induces apoptosis by escalating the level of the CCAAT/enhancer-binding protein homologous protein (CHOP) (Irie et al., 2011; Takeichi et al., 2012). In addition, microglial activation and METH-mediated neuroinflammation also contribute to neurotoxicity by attacking the neuron with inflammatory cytokines such as tumor necrosis factor (TNF)-α and interleukin (IL)-6 (Coelho-Santos et al., 2012). Thus, METH can cause terminal damage and neurodegeneration of the neuronal cells in the hippocampus, eventually resulting in cognitive disorder.

The ever-increasing levels of METH abuse have necessitated the development of effective intervention strategies that protect neural cells against METH-induced neurotoxic consequences (Yang et al., 2018). Molecular hydrogen, which is characterized by its anti-oxidative, anti-inflammatory, and anti-apoptotic properties, has recently gained attention as a potential novel healthcare product for preventive and therapeutic applications in a wide range of pathological conditions (Fujita et al., 2009; Ohta, 2012; Spulber et al., 2012). Several studies have demonstrated the protective effects of molecular hydrogen in various CNS disease animal models, such as stroke, traumatic brain injury, Alzheimer’s disease, and Parkinson’s disease (Fu et al., 2009; Dohi et al., 2014; Ono et al., 2017; Nishimaki et al., 2018). Our previous study found that hydrogen-rich saline (HRS) injections significantly attenuated naloxone-precipitated morphine withdrawal symptoms and morphine withdrawal-induced anxiety-like behaviors as well as elevated corticosterone levels (Wen et al., 2017). A recent study also showed that consumption of hydrogen-rich water (HRW) prevented stress-induced impairments in learning tasks during chronic physical restraint by buffering the effect of oxidative stress (Nagata et al., 2009). It’s encouraging that a multicenter randomized trial is underway to confirm the efficacy of molecular hydrogen on neurological outcomes in patients with post-cardiac arrest syndrome (Tamura et al., 2017). Some methods have also been used to administer hydrogen to humans, including drinking or bathing with HRW and inhalation of hydrogen gas (HG). HRW drinking is expected to be easily used in daily life, and the results of several clinical studies of HRW were recently released (Kang et al., 2011; Iida et al., 2016; Korovljev et al., 2019). Herein, we hypothesized that the consumption of HRW may potentially exert a preventive effect on METH-induced neurotoxicity and spatial learning and memory impairments.

In the current study, we have performed the Barnes maze and Morris water maze tests to investigate the effects of *ad libitum* HRW consumption on high-dose METH exposure-induced spatial learning task impairment and memory loss. The neuronal damage was evaluated by Nissl staining, and the index of mitochondrial dysfunction, ER stress, and neuroinflammation were also measured by the detection of Bax/Bcl-2, caspase 3, GRP78, CHOP and p-NF-κB p65 expression, and IL-6 and TNF-α level in hippocampus.

## Materials and Methods

### Animals

Male C57BL/6 mice, initially weighing 20–22 g (8 weeks old), were purchased from Beijing Vital River Laboratory Animal Technology Co. Ltd, China. All the mice were acclimated to the laboratory housing conditions for 5 days before the experiment. The animals were housed in a climate-controlled environment. Constant temperature (21 ± 2°C), humidity (approximately 60%), and a 12-h light/dark cycle (lights on at 7:00 am) were maintained. Food and water were available *ad libitum*.

### Drugs

DL-METH was provided by Beijing Municipal Public Security Bureau, China. The stock solution of METH (1 g/ml) was prepared in saline. The concentration of METH was adjusted to an appropriate injection volume of 10 ml/kg of body weight immediately before use. HRW was provided by Huoli Qingyuan Biotechnology Co. Ltd., Beijing, China, and stored under atmospheric pressure at ambient temperature in an aluminum pot without dead volume. The concentration of HRW was maintained at approximately 2.0 mg/L in this study.

### Induction of METH-Induced Neurotoxicity and *Ad Libitum* HRW Consumption

To explore the effects of *ad libitum* HRW consumption on METH-induced neurotoxicity and spatial learning and memory impairment, the mice were administered to four METH intraperitoneal injections (10 mg/kg at a 3-h interval). Stereotypic behavior and body temperature were tested after the last METH injection. After the behavioral assessments, the mice were put back into their home cages and consumed HRW *ad libitum* for 7 days. The HRW bottles were changed every 12 h.

### Behavioral Tests

#### Stereotypic Behavior

Stereotypic behaviors were observed and scored as described by Sams-Dodd (1998) after the last injection of METH in mice. The mouse was placed in the test apparatus to assess its stereotypic behavior for 1 h. The behavior was assessed at 10-min intervals, and the total scores for each mouse were calculated.

#### Barnes Maze

The Barnes maze was a circular platform (90 cm in diameter) with 20 holes (5 cm in diameter) around the perimeter (JLBeh Soft-tech Co. Ltd., Shanghai, China). A removable escape box was placed beneath the target hole. Fixed maze visual cues were hung above the four quadrants around the maze. Overhead lights provided motivation for the animal to seek the escape box. The motions of the animal were recorded by a camera above the center of the maze.

The behavioral assessment (**Figure 3A**) was performed according to the study by McQuown et al. (2019). The mice were given a free exploration trial for adaptation by placing the subject directly in front of the target position and guiding the animal into the escape box on day 9. The training procedure included two trials per day on days 10–13. At the start of each training trial, mice were placed inside a start tube at the center of the platform for 15 s and then released. After entering the escape box, the mice remained in the escape box for 30 s before returning to the home cage. The escape latency was recorded and scored as measures of acquisition. The maze and escape box were cleaned with 70% ethanol solution to remove odor cues. A probe test consisting of a 120-s free exploration period without the escape box was conducted on day 14. The exploration time spent in the target quadrant and the total traveled distance were determined. The escape latency, exploration time spent in the target quadrant, and total traveled distance were measured automatically by using the Animal Video Analysis System (JLBeh Soft-tech Co. Ltd., Shanghai, China).

#### Morris Water Maze

The water maze was a black circular pool (120 cm in diameter and 50 cm in height), filled with 21 ± 1°C water to a depth of 20 cm (JLBeh Soft-tech Co. Ltd., Shanghai, China). A hidden circular platform (6 cm in diameter) was in the center of the target quadrant, submerged 1.0 cm beneath the surface of the water. Fixed maze visual cues were hung above the four quadrants around the maze. The motions of the animal were recorded by a camera above the center of the maze.

The behavioral assessment (**Figure 4A**) was conducted as in our previous study (Yang et al., 2013). The mice were given a 60-s free swimming trial for adaptation in the pool without the platform on day 15. The training procedure included four trials per day with four different starting positions on days 16–19. In each trial, animals were given a maximum of 60 s to find the platform. After mounting the platform, the mice remained on the platform for 30 s. The escape latency was recorded and scored as measures of acquisition. A probe trial consisting of a 60-s free swim period without the platform was performed to test spatial memory on day 20. The swimming time spent in each quadrant and swimming speed were recorded. The escape latency, time spent in each quadrant, and swimming speed were measured automatically by using the Animal Video Analysis System (JLBeh Soft-tech Co. Ltd., Shanghai, China).

### Nissl Staining

Morphological alterations in the hippocampus after METH exposure were detected by performing Nissl staining. Mice were killed, and their brains were harvested after 7 days of *ad libitum* HRW consumption. The tissue samples were embedded in paraffin, and 5-mm-thick sagittal sections were prepared for staining. The hippocampus area (Bregma -1.58 mm∼-2.06 mm) was accurately identified according to the stereotaxic atlas (Paxinos and Franklin, 2001). Using the serial section technique, one of every five sections was selected for a total of three sections from each mouse. The slides were stained in 1% cresyl violet solution for 10 min and rinsed quickly in distilled water. Then, they were mounted with a resinous medium and assessed microscopically. Normal neurons showed regular cell morphology and round nuclei. Damaged neurons showed an irregular cell body, shrinking and hyperchromatic nucleus, and dried-up cytoplasm with vacuoles. Cell counting was performed at 400× magnification image. The average number of damaged neurons in CA1 and CA3 areas was calculated by two independent observers who were blinded to the experimental conditions. The data for each mouse were derived from the average of those three sections.

### Enzyme-Linked Immunosorbent Assay

The levels of ΙL-6 and TNF- α in the hippocampus were detected by enzyme-linked immunosorbent assay (ELISA). Supernatants from the hippocampal tissues of each group were collected and stored at −80°C until analysis. Measurements using the ELISA Kit (EMC004 and EMC102a, Neobioscience, Shenzhen, China) were performed according to the manufacturer’s instructions.

### Western Blot

The hippocampal tissues were harvested at scheduled time points to test the expression levels of proteins indicating mitochondrial dysfunction and ER stress, including Bax, Bcl-2, caspase 3, GRP78, and CHOP. Equal amounts of protein from each sample were separated by using 10% sodium dodecyl sulfate polyacrylamide gel electrophoresis (SDS-PAGE). The separated proteins were transferred onto nitrocellulose membranes. The blots were blocked with 5% non-fat dry milk in TBS solution and probed with Bax (ab32503), Bcl-2 (ab185002), caspase 3 (ab197202), GRP78 (ab21685), CHOP (ab11419), and p-NF-κB p65 (ab222494) antibodies (diluted 1:1,000, Abcam) overnight at 4°C. The membranes were then washed and incubated with a fluorophore-conjugated donkey anti-rabbit secondary antibody (diluted 1:10,000, Rockland), which underwent excitation with light (700/800 nm). The emitted light was then detected and analyzed by using an Odyssey gel imaging system (LI-COR, Inc., Lincoln, NE, USA). β-Actin expression was analyzed in the same blots by using a monoclonal antibody (BS6007M, diluted 1:1500; Bioworld Technology). Relative expression was quantified with respect to the signals of the corresponding β-actin band.

### Experimental Design

**Figure 1:**
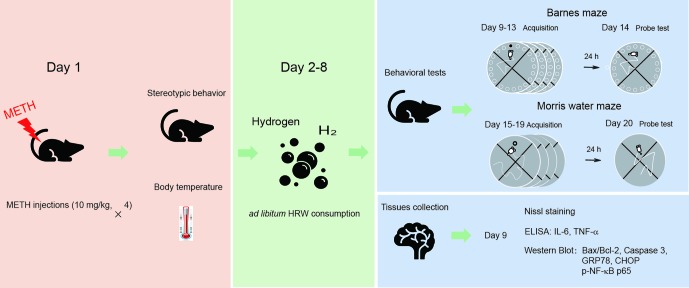
The experimental procedure and drug treatment. The mice were treated with methamphetamine (METH; 10 mg/kg × 4-, 3-h interval), and the naive controls were treated with an equal number of saline injections. Stereotypic behavior and body temperature were observed immediately after the last METH injection. When the mice were returned to their home cages, they were allowed to consume water or hydrogen-rich water (HRW) *ad libitum* for 7 days (days 2–8). On days 9–14 and 15–20, a subgroup of mice from each group performed the Barnes maze and Morris water maze tests. The remaining mice in each group were decapitated, and their brains were harvested for Nissl staining, Western blot, and enzyme-linked immunosorbent assay (ELISA) on day 9.

The experimental procedure and drug treatment are shown in **Figure 1**. The mice were randomly assigned to one of four groups (18 mice per group). Two groups of mice were treated with METH (4 × 10 mg/kg, 3-h interval), and the other two groups were naive controls that were treated with an equal number of saline injections. Stereotypic behavior and body temperature were initially observed to evaluate the effect of high-dose METH exposure (day 1). When the mice in all four groups were returned to their home cages after METH treatment and behavioral observation, they received water or HRW *ad libitum* for 7 days (days 2–8). The following experiments were subsequently performed: I) to assess the possible effect of *ad libitum* HRW consumption on high-dose METH exposure-induced spatial learning task impairment and memory loss, the Barnes maze and Morris water maze tests were performed with 10 mice in each group on days 9–14 and days 15–20, and II) four mice in each group were perfused with 4% paraformaldehyde and their brains were harvested for Nissl staining, and the remaining four mice in each group were decapitated and their hippocampal tissues were collected for Western blot analysis and ELISA on day 9. The degree of mitochondrial dysfunction, ER stress, and neuroinflammation were measured by evaluating Bax/Bcl-2, caspase 3, GRP78, CHOP, and p-NF-κB p65 expression and IL-6 and TNF-α levels in the hippocampus.

### Data Analysis

The data are presented as means ± SEM. The results of the Morris water maze and Barnes maze tests were analyzed using repeated-measures analysis of variance, with the test phase as the within-subjects factor and the drug treatments as the between-subjects factor. The scores for stereotypic behavior and body temperature were analyzed by Student’s *t* test. The results of exploration time and motion speed of mice in the probe test, Nissl staining, and molecular targets (Western blot and ELISA) were analyzed by using two-way analysis of variance (ANOVA). *Post hoc* comparisons were performed by using the Bonferroni test. The criterion for statistical significance was *p* < 0.05 (SPSS, v.16.0, Chicago, USA).

## Results

### Animal Exclusion

The group sizes were unequal due to accidental deaths of animals after high-dose METH exposure and exclusions due to poor general activity in the behavioral tests. In total, three mice died after the last METH injection, and two mice (one in METH group and one in HRW group) were excluded from the statistical analysis because of low mobility in behavioral test. A total of 67 animals were evaluable in this study.

### METH-Induced Stereotypic Behavior and Hyperthermia

**Figure 2:**
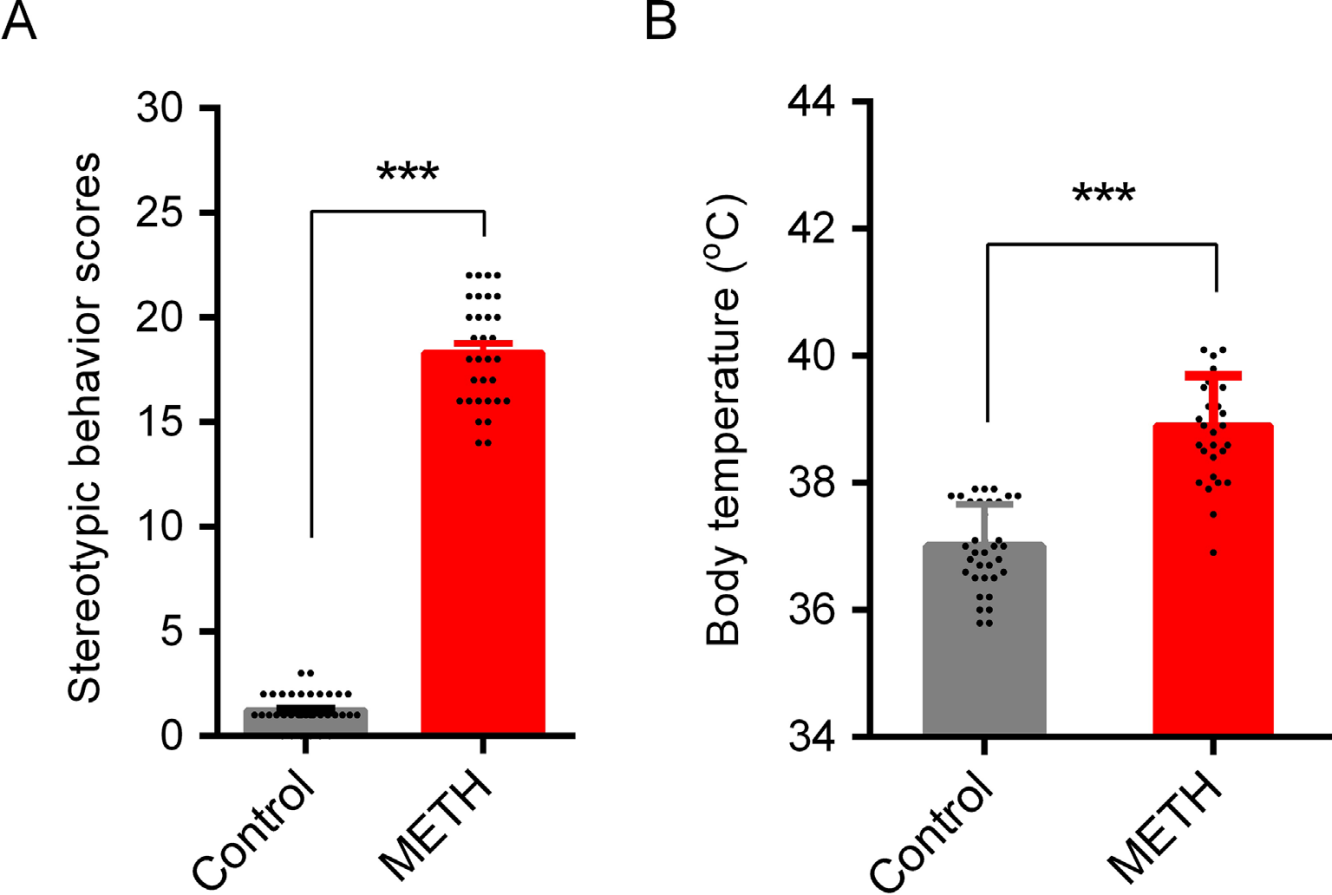
METH-induced stereotypic behavior and hypothermia in mice. Stereotypic behavior **(A)** and body temperature **(B)** were tested after the last injection of METH. One animal in control group (subsequent HRW group) and one in METH group (subsequent METH group) were excluded because of low mobility in behavioral test, and three mice in METH group were died after the last METH injection. Data are expressed as the mean ± SEM; n = 35 for the control group, and n = 32 for the METH group; and ***p < 0.001, compared to the control group.

Stereotypic behaviors and hyperthermia were induced by METH exposure. As shown in **Figure 2**, repeated high-dose METH treatment significantly increased the total Sams-Dodd scores (t = 38.40, *p* < 0.001; **Figure 2A**) and body temperature (t = 10.72, *p* < 0.001; **Figure 2B**). Saline- or METH-treated mice were returned to their home cages for the subsequent *ad libitum* water or HRW consumption treatment.

### *Ad libitum* HRW Consumption Inhibited METH-Induced Spatial Learning Task Impairment and Memory Loss

#### Barnes Maze

**Figure 3:**
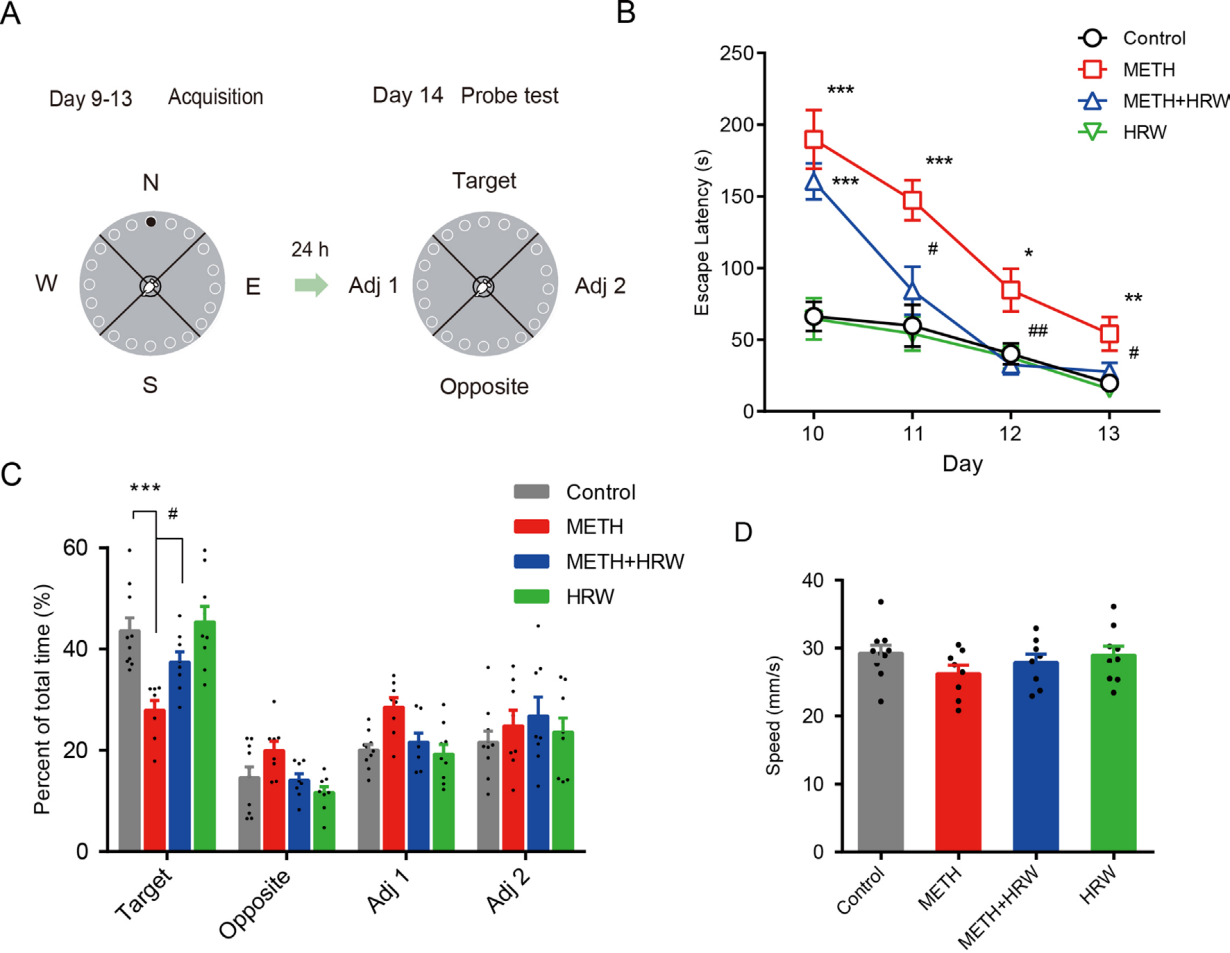
Effects of *ad libitum* HRW consumption on METH-induced spatial learning task impairment and memory loss in the Barnes maze test. **(A)** Experimental procedure for the Barnes maze test. **(B)** The escape latencies of mice in the spatial learning task on four training days. **(C)** The exploration time (percent of total time) spent in the four quadrants (target, opposite, adjacent 1, and adjacent 2) during the probe test. **(D)** Motion speed of mice in the probe test. Data are expressed as the mean ± SEM; n = 10, 8, 8, and 9, respectively; *p < 0.05, **p < 0.01, ***p < 0.001, compared to the control group; and ^#^ *p* < 0.05, ^##^ *p* < 0.01 compared to the METH group.

In the 4-day training period for the Barnes maze test, high-dose METH treatment was shown to significantly affect the performance in the spatial learning task (**Figure 3B**). Analysis using repeated-measures with escape latency revealed a significant effect of METH treatment (F_(1,16)_ = 41.81, *p* < 0.001) and time (F_(3,48)_ = 26.93, *p* < 0.001) and the interaction between METH treatment and time (F_(3,48)_ = 6.809, *p* < 0.001). However, *ad libitum* HRW consumption inhibited the METH-induced spatial learning task impairment in the Barnes maze test. A significant effect of time (F_(3,45)_ = 28.32, *p* < 0.001) and *ad libitum* HRW consumption treatment (F_(1,15)_ = 18.50, *p* < 0.001) and the interaction between *ad libitum* HRW consumption treatment and time (F_(3,45)_ = 8.384, *p* < 0.001) was observed.

On the probe-test day, the percentages of exploration time spent in the four quadrants (**Figure 3C**) and motion speed (**Figure 3D**) were recorded. ANOVA with percentage of exploration time spent in the target quadrant revealed a significant effect of METH (F_(1,31)_ = 24.930, *p* < 0.001) and *ad libitum* HRW consumption treatment (F_(1,31)_ = 6.286, *p* = 0.018), but no significant effect of the interaction (F_(1,31)_ = 3.291, *p* = 0.079) between METH and *ad libitum* HRW consumption treatment. The Bonferroni *post hoc* test indicated that high-dose METH exposure resulted in memory loss and decreased the time spent in the target quadrant (*p* < 0.001), and *ad libitum* HRW consumption inhibited the METH-induced memory loss in the Barnes maze probe test (*p* < 0.05). Moreover, two-way ANOVA with motion speed revealed no significant effects of METH (F_(1,31)_ = 2.573, *p* = 0.119), *ad libitum* HRW consumption (F_(1,31)_ = 0.269, *p* = 0.608) treatment, and the interaction between these two factors (F_(1,31)_ = 0.578, *p* = 0.453).

#### Morris Water Maze

**Figure 4:**
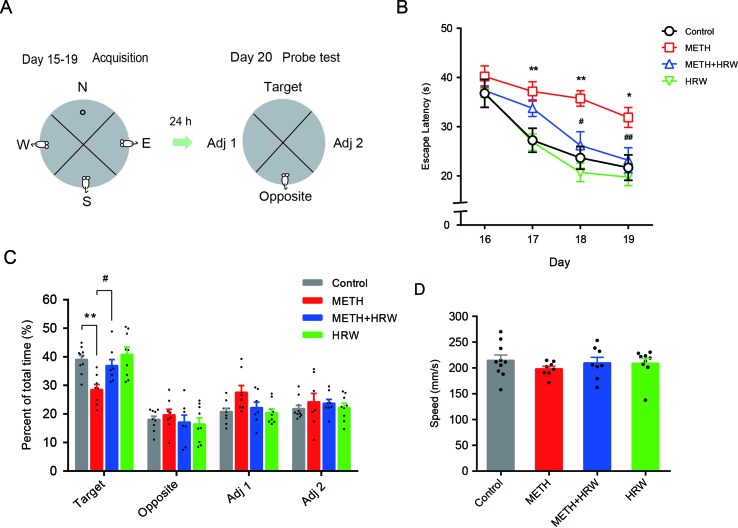
Effects of *ad libitum* HRW consumption on METH-induced spatial learning task impairment and memory loss in the Morris water maze test. **(A)** Experimental procedure for the Morris water maze test. **(B)** The average escape latencies of four trials for each mouse in the spatial learning task on four training days. **(C)** The swimming time (percent of total time) spent in the four quadrants (target, opposite, adjacent 1, and adjacent 2) during the probe test. **(D)** Swimming speed of mice in the probe test. Data are expressed as the mean ± SEM; n = 10, 8, 8, and 9, respectively; *p < 0.05, **p < 0.01 compared to the control group; and ^#^ *p* < 0.05, ^##^ *p* < 0.01 compared to the METH group.

The effects of *ad libitum* HRW consumption on METH-induced spatial learning task impairment and memory loss were further confirmed by the Morris water maze test. As shown in **Figure 4B**, the analysis of repeated-measures with escape latency in the control and METH groups revealed a significant effect of METH treatment (F_(1,16)_ = 12.960, *p* = 0.002) and time (F_(3,48)_ = 14.370, *p* < 0.001), but no significance in the interaction between METH treatment and time (F_(3,48)_ = 2.067, *p* < 0.117), as well as a significant effect of *ad libitum* HRW consumption (F_(1,14)_ = 12.130, *p* = 0.004) and time (F_(3,42)_ = 13.940, *p* < 0.001), but no significance in the interaction between *ad libitum* HRW consumption and time (F_(3,42)_ = 1.743, *p* = 0.173) in the METH and METH + HRW groups.

Subsequent analysis of probe-test data by using two-way ANOVA with swimming time spent in the target quadrant (**Figure 4C**) indicated a significant effect of METH (F_(1,31)_ = 12.960, *p* = 0.001) and *ad libitum* HRW consumption (F_(1,31)_ = 6.393, *p* = 0.017) treatment, but no significant effect of the interaction (F_(1,31)_ = 2.788, *p* = 0.105) between METH and *ad libitum* HRW consumption treatment. The Bonferroni *post hoc* test indicated a decrease in the swimming time spent in target quadrant in METH-treated mice (*p* < 0.01) and a recovery after the *ad libitum* HRW consumption (*p* < 0.05). Two-way ANOVA with swimming speed (**Figure 4D**) excluded the possibility of drug interference with animal motor coordination and revealed no significant effects of METH (F_(1,31)_ = 0.687, *p* = 0.414), *ad libitum* HRW consumption (F_(1,31)_ = 0.097, *p* = 0.758) treatment, and the interaction between these two factors (F_(1,31)_ = 0.807, *p* = 0.376). These findings indicated that *ad libitum* HRW consumption after METH exposure significantly improved the performance in the spatial learning task and inhibited METH-induced memory loss.

### *Ad Libitum* HRW Consumption Rescued METH-Induced Neuronal Damage in the Hippocampus

**Figure 5:**
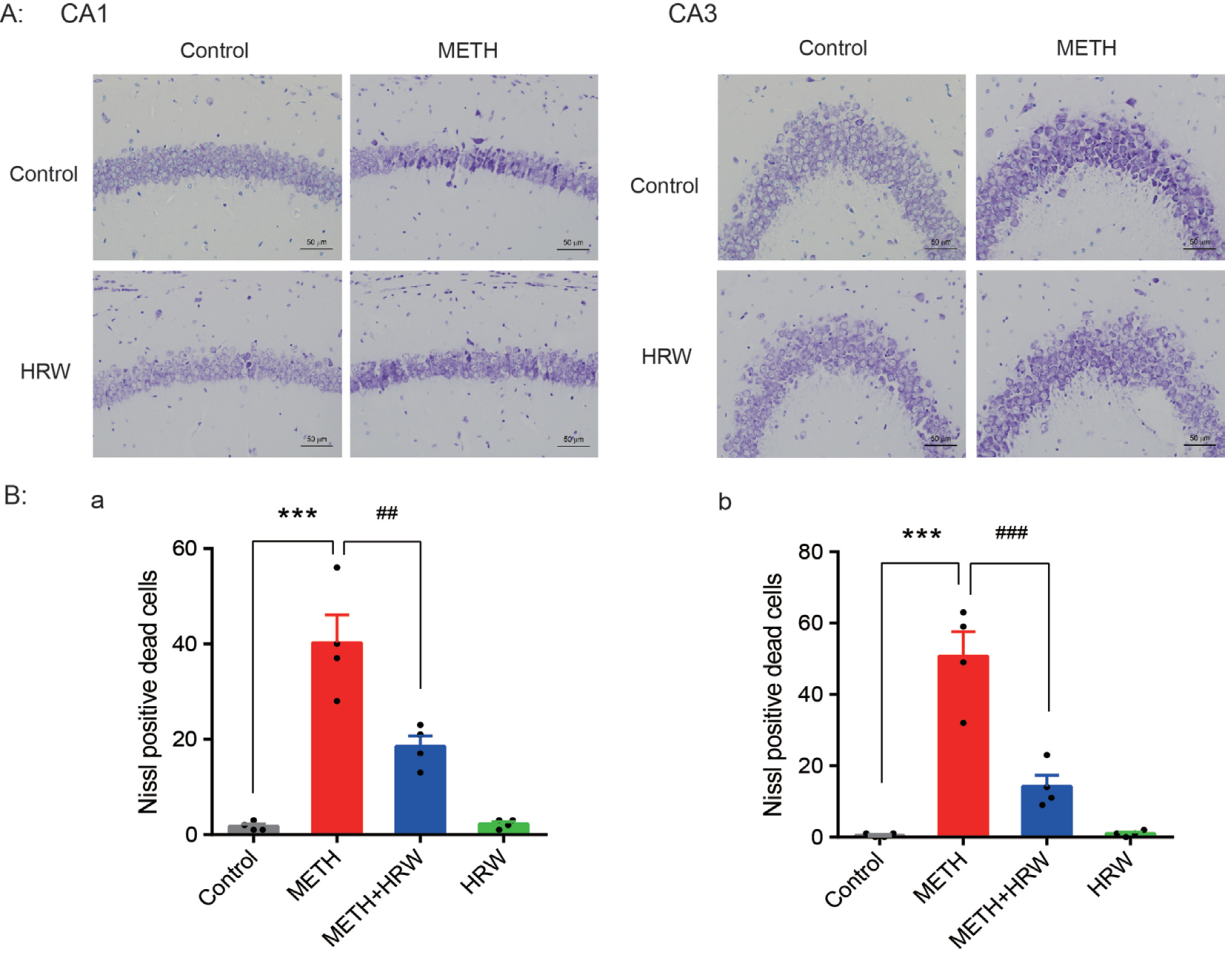
Effects of *ad libitum* HRW consumption on METH-induced neuronal damage in the hippocampus. **(A)** Representative photomicrograph of Nissl staining. Bars = 50 mm. **(B)** Numbers of Nissl-positive dead cells in the CA1 (a) and CA3 (b) regions of the hippocampus. Data are expressed as the mean ± SEM; n = 4 for each group; ***p < 0.001 compared to the control group; and ^##^ *p* < 0.01, ^###^ *p* < 0.001 compared to the METH group.

Nissl staining was used to confirm structural atrophy and explore the effect of *ad libitum* HRW consumption on METH-induced neuronal damage in the hippocampus (**Figure 5A**). Two-way ANOVA with the number of Nissl-positive dead cells in CA1 and CA3 revealed a significant effect of METH (F_(1,12)_ = 76.01, *p* < 0.001; F_(1,12)_ = 70.08, *p* < 0.001, respectively) and *ad libitum* HRW consumption (F_(1,12)_ = 11.45, *p* = 0.005; F_(1,12)_ = 22.52, *p* < 0.001, respectively), and the interaction (F_(1,12)_ = 12.55, *p* = 0.004; F_(1,12)_ = 23.79, *p* < 0.001, respectively) between METH and *ad libitum* HRW consumption. The results of Bonferroni *post hoc* test showed that the number of damaged neurons in the hippocampal CA1 (**Figure 5B-a**) and CA3 (**Figure 5B-b**) regions significantly increased following METH injection in comparison with the control group (*p* < 0.001), and these alterations were significantly attenuated by *ad libitum* HRW consumption in comparison with the METH group (*p* < 0.01 and *p* < 0.001 for CA1 and CA3, respectively).

### *Ad libitum* HRW Consumption Reversed METH-Induced Changes in the Degree of Mitochondrial Dysfunction, ER Stress, and Neuroinflammation

**Figure 6:**
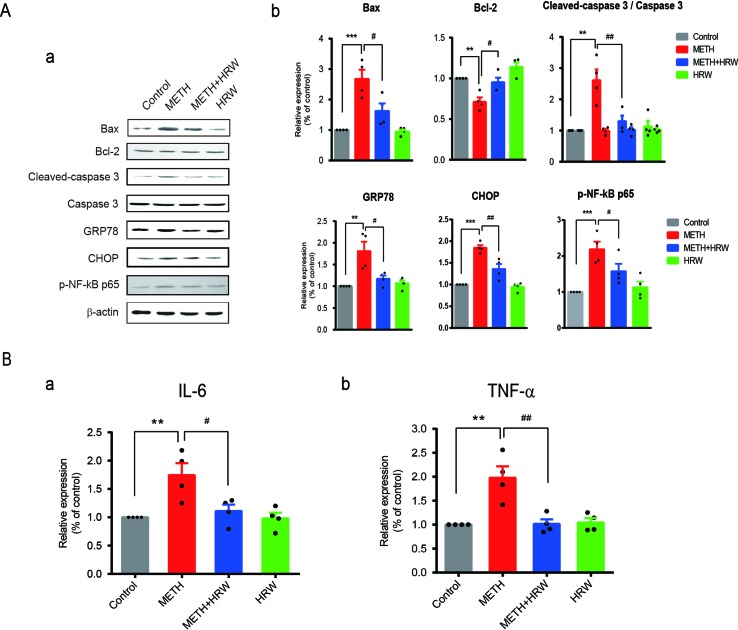
Effects of *ad libitum* HRW consumption on METH-induced changes in the indicators of mitochondrial dysfunction, endoplasmic reticulum stress (ERS), and neuroinflammation. **(A)** Western blot analysis of Bax, Bcl-2, cleaved caspase 3, caspase 3, GRP78, CHOP, and p-NF-κB p65 expression in the hippocampus. **(B)** Levels of IL-6 **(a)** and TNF-α **(b)** in the hippocampus determined by ELISA. Data are expressed as the mean ± SEM; n = 4 for each group; **p < 0.01, ***p < 0.001 compared to the control group; and ^#^ *p* < 0.05, ^##^ *p* < 0.01 compared to the METH group.

Alterations in the degree of mitochondrial dysfunction, ER stress, and neuroinflammation in all groups are shown in **Figure 6**. The expression levels of Bax, Bcl-2, cleaved caspase 3, caspase 3, GRP78, CHOP, and p-NF-κB p65 (**Figure 6A**) in the hippocampus were quantified by using Western blotting, and the IL-6 (**Figure 6B-a**) and TNF-α levels (**Figure 6B-b**) were detected by ELISA. Two-way ANOVA revealed a significant effect of METH (Bax: F_(1,12)_ = 36.050, *p* < 0.001; Bcl-2: F_(1,12)_ = 25.53, *p* < 0.001; cleaved caspase 3: F_(1,12)_ = 17.18, *p* = 0.001; GRP78: F_(1,12)_ = 15.02, *p* = 0.002; CHOP: F_(1,12)_ = 81.23, *p* < 0.001; p-NF-κB p65: F_(1,12)_ = 28.87, *p* < 0.001; IL-6: F_(1,12)_ = 11.39, *p* = 0.006; and TNF-α: F_(1,12)_ = 13.66, *p* = 0.003) and *ad libitum* HRW consumption (Bax: F_(1,12)_ = 7.885, *p* = 0.016; Bcl-2: F_(1,12)_ = 16.350, *p* = 0.002; cleaved caspase 3: F_(1,12)_ = 7.547, *p* = 0.018; GRP78: F_(1,12)_ = 5.777, *p* = 0.033; CHOP: F_(1,12)_ = 14.940, *p* = 0.002; p-NF-κB p65: F_(1,12)_ = 5.315, *p* = 0.039; IL-6: F_(1,12)_ = 6.331, *p* = 0.027; and TNF-α: F_(1,12)_ = 11.230, *p* = 0.006), and the interaction between METH and *ad libitum* HRW consumption treatment (Bax: F_(1,12)_ = 6.556, *p* = 0.025; cleaved caspase 3: F_(1,12)_ = 11.320, *p* = 0.006; GRP78: F_(1,12)_ = 9.056, *p* = 0.011; CHOP: F_(1,12)_ = 9.877, *p* = 0.009; p-NF-κB p65: F_(1,12)_ = 8.475, *p* = 0.013; IL-6: F_(1,12)_ = 5.656, *p* = 0.035; and TNF-α: F_(1,12)_ = 11.990, *p* = 0.005). However, the interaction of the two factors showed no significant effect on the expression of Bcl-2 (F_(1,12_) = 1.072, *p* = 0.321). The Bonferroni *post hoc* test indicated an increase in Bax, cleaved caspase 3, CHOP, p-NF-κB p65 (*p* < 0.001), and GRP78 (*p* < 0.01) expression and IL-6 and TNF- α secretion (*p* < 0.01), but a decrease in Bcl-2 (*p* < 0.01) expression in METH-treated mice. *Ad libitum* HRW consumption significantly reversed the METH-induced changes in the above parameters (*p* < 0.05 for Bax, Bcl-2, GRP78, and p-NF-κB p65; *p* < 0.01 for cleaved caspase 3, CHOP, and TNF-α).

## Discussion

This study aimed to check if consumption of HRW may potentially exert a preventive effect on METH-induced neurotoxicity and spatial learning and memory impairments. Herein, we present the first set of findings demonstrating a significant inhibitory effect of molecular hydrogen delivered by *ad libitum* HRW consumption on the METH-induced spatial learning impairment and memory loss noted in the Barnes maze and Morris water maze tests. We also found that *ad libitum* HRW consumption significantly restrained neuronal damage in the hippocampus after high-dose METH exposure. Treatment with *ad libitum* HRW consumption also had an inhibitory effect on METH-induced mitochondrial dysfunction, ER stress, and neuroinflammation. Therefore, our study may pave the way for a novel and effective neuroprotective strategy using molecular hydrogen for METH-induced neurotoxicity.

Illicit drug abuse is a serious global problem. Among the current illicit drugs, METH is the most prevalent illicit drug in numerous countries, including China (Bramness et al., 2015; Kwon and Han, 2018; Bannwarth et al., 2019). METH abuse has been shown to result in cognitive deficits in attention, learning, and memory in a variety of experimental and clinical studies (Potvin et al., 2018; Seyedhosseini Tamijani et al., 2018). Several dose regimens of METH administration have been evaluated in different studies, such as a single high dose (40 mg/kg) or repeated (4 × 10 mg/kg, 2–3-h intervals) or escalating (1–10 mg/kg, twice a day, at 5-h intervals, for 10 days) doses of METH and chronic voluntary oral METH intake (Yang et al., 2018). Since some studies have suggested that exposure to nontoxic amounts of METH can afford protection against the toxic effects of subsequent larger doses, a phenomenon referred to as METH preconditioning, a regimen based on 4 × 10 m/kg METH injections spaced 3 h apart, was used in mice in the present study. Stereotypic behavior and hyperthermia were obviously induced after drug administration. After METH exposure, two different behavioral tests were utilized to analyze and confirm the efficiency of molecular hydrogen. The Barnes maze and Morris water maze tests are both common behavioral models that are valuable for evaluating spatial learning and memory ability of rodents. It’s worthy to note that behavioral tasks involving high levels of stress can influence the animal’s performance on the task. Strong aversive stimulus induced by swimming in the Morris water maze might be a factor account for the different learning curves in this study. Consistent with the results of previous studies (Vorhees, 1997; Gutierrez et al., 2018), the findings in both behavioral tests revealed spatial learning task impairment and memory loss at 7 days after drug exposure in the METH-treated mice. Moreover, METH exposure caused damage to the pyramidal neurons in the CA1 and CA3 regions of the hippocampus, which play crucial roles in cognition. In fact, we also tested METH-induced neuronal damage in dentate gyrus (DG). However, the inconsistent result was revealed, and the damage was only observed in one animal. The reason might be complicated, and we could not regard it as a negative region. Observations in more samples were needed in the following study.

Considering the primary mechanisms underlying the effects of METH, reducing oxidative damage or neuroinflammation may be feasible approaches to prevent METH-induced neurotoxicity. Molecular hydrogen was assumed to be effective in relieving the pathologic changes after METH exposure. In this study, we found that molecular hydrogen significantly inhibited the METH-induced spatial learning impairment and memory loss and hippocampal neuronal damage. Injections of HRS and inhalation of HG are common strategies to rapidly deliver molecular hydrogen into experimental animals and the human body. However, these approaches are not practical or suitable for continuous consumption in daily life. Drinking HRW is clearly much more convenient. In our experiments, *ad libitum* HRW consumption, a method that fits well within the normal lifestyle of humans, was applied to administer hydrogen to animals, and it had significant neuroprotective effects on METH-induced neurotoxicity. As for the concentration of hydrogen, we attempted to measure the hydrogen concentrations in the blood and brain homogenate after intraperitoneal injection of HRS by performing cyclic voltammetry analysis using a hydrogen electrode. We could detect a fivefold and threefold change in the hydrogen concentrations in the blood and brain homogenate, respectively, at 5 min after HRS injection (unpublished data not shown). However, since this test could not be reliably conducted on free-moving animals in the present condition of our lab, we did not test the hydrogen concentration in the blood and brain of mice that consumed HRW *ad libitum*. Nevertheless, one recent study has reported that the blood concentration of hydrogen incorporated from the stomach of animals consuming HRW was approximately 5 µM (the control data was 1 µM) (Nagata et al., 2009). Since hydrogen can quickly reach the maximal serum concentration and easily cross the blood–brain barrier, we considered that hydrogen had been incorporated into brain tissues after *ad libitum* HRW consumption.

Oxidative stress, the most significant factor contributing to METH-induced neurotoxicity, leads to mitochondrial dysfunction and ER stress. It eventually results in devastating neuropathological effects and neuronal death in the brain due to the subsequent Bax/Bcl-2 changes in the mitochondrial fractions and caspase activation, as well as the accumulation of misfolded proteins and elevation of GRP78 and CHOP expression (Shah and Kumar, 2016; Qie et al., 2017). Similar results were obtained in METH-treated mice in the present study. Furthermore, METH activates NF-kB signaling pathway and promoting the transcription of pro-inflammatory cytokines in the brain after 3 to 7 days of METH treatment (Goncalves et al., 2010), which corroborate our data showing a late increase in the levels of markers characteristic of neuroinflammation, such as p-NF-kB p65, and the pro-inflammatory cytokines IL-6 and TNF-α. In addition, HRW significantly inhibited the increase in Bax/Bcl-2, cleaved caspase-3, GRP78, CHOP, and p-NF-kB p65 levels and decreased the amounts of IL-6 and TNF-α in the hippocampus of METH-treated mice. This indicates that molecular hydrogen attenuated the METH-induced mitochondrial dysfunction, ER stress, and neuroinflammation in the hippocampus.

However, the lack of animal general condition record such as change of body weight after large dose of METH treatment was a limitation of this study. Moreover, METH abuse leads to dysfunction of several brain regions, and most of the studies have been extensible exploring the toxic effects of METH in the striatum (Gou et al., 2015; Xie et al., 2018; Sabrini et al., 2019). In view of this point, we planned to observe the effects of hydrogen on the DA transporter activity and striatal dopaminergic terminal damage in the animal model by using chronic repeated METH treatment regimens in a following study. Although we confirmed the efficiency of HRW consumption on METH-induced neurotoxicity, the main mechanisms underlying the inhibitory effect of molecular hydrogen have not been clearly clarified. The effects of hydrogen on early upregulation of markers characteristic of oxidative damage after METH exposure, such as ROS and malondialdehyde (MDA), and the roles of hydrogen over glia cells need to be explored in future studies.

## Conclusion

In summary, the present investigation showed that *ad libitum* HRW consumption significantly attenuated METH-induced spatial learning impairment and memory loss as well as the neuronal damage in hippocampus. Since reversing or preventing the oxidative damage and neuroinflammation induced by METH exposure might be useful in protecting against neuronal damage, molecular hydrogen may have a potential application in reducing the risk of neurodegeneration frequently observed in METH abusers.

## Data Availability

The datasets analyzed in this manuscript are not publicly available. Requests to access the datasets should be directed to wendi01125@126.com.

## Ethics Statement

All experiments were conducted according to the guidelines of the National Institutes of Health Guide for the Care and Use of Laboratory Animals. The Local Committee on Animal Care and Use and Protection of the Hebei Medical University approved the experimental procedures.

## Author Contributions

CM, BC, and DW conceived this work. DW wrote the main manuscript text. DW and RH performed the lab experiments. RH, JW, and XS performed the animal experiments. MG performed the Nissl staining. BX and FY analyzed and interpreted the data. All authors read and commented on the manuscript.

## Funding

This study was financially supported by the Science and Technology Research Projects for University in Hebei Province (No. BJ2017015).

## Conflict of Interest Statement

The authors declare that the research was conducted in the absence of any commercial or financial relationships that could be construed as a potential conflict of interest.
